# On the Pose Estimation of an Untethered Microrobot via Magnetic Localization and Optical Tracking

**DOI:** 10.3390/mi17101196

**Published:** 2026-10-16

**Authors:** Tuğrul Uslu, Erkin Gezgin

**Affiliations:** 1 Department of Mechanical Engineering, İzmir Katip Çelebi University, 35620 İzmir, Türkiye; 2 Department of Mechatronics Engineering, İzmir Katip Çelebi University, 35620 İzmir, Türkiye; erkin.gezgin@ikcu.edu.tr

**Keywords:** microrobotics, surgical navigation, motion capture, registration, hybrid tracking

## Abstract

The field of medical microrobotics has emerged as a promising approach for delicate medical interventions in anatomical regions where conventional approaches lack efficiency. Due to the fact that constrained anatomical workspaces offer limited visibility, in order to fulfill desired tasks, the precise tracking of untethered microrobots becomes a vital necessity and a critical challenge. Although there are studies in the literature to overcome said challenge, drawbacks include but are not limited to prolonged exposure to high magnetic fields, limited tracking workspace, and robustness. In light of this, the current paper proposes a hybrid tracking system utilizing both optical and magnetic tools to provide real-time pose estimation of an untethered microrobot operated within a closed workspace. The proposed system utilizes a custom-made Hall-effect sensor array apparatus that is capable of single-degree-of-freedom actuation to execute a planar sweeping motion and three low-cost motion capture cameras to track the apparatus pose in real time. In combining precise optical tracking, magnetic sensing, and the proposed artificial neural network (ANN)-based estimation approach, the system overcomes visibility limitations of optical motion tracking systems and workspace limitations in magnetic sensing systems. Hardware verification studies of the constructed setup are carried out utilizing a KUKA KR6 R900 SIX serial robot manipulator to simulate untethered microrobot actuation. During verification procedures, the sensor apparatus motion, end effector of the manipulator, and microrobot are streamed into a 3D Slicer software virtual environment to enhance visual feedback with an efficient surgical navigation approach. The proposed approach shows efficient results and sub-millimeter accuracy in microrobot tracking.

## 1. Introduction

The field of medical microrobotics has emerged as a promising approach for targeted interventions, like drug delivery in hard-to-access anatomical regions, including the eye [1], the vascular system [2], cochlear canals [3] and cerebral aneurysms [4]. Unlike traditional tethered surgical tools, untethered microrobots provide better maneuverability in the anatomical workspace and minimize tissue damage. In order to effectively perform microrobot operations in these spaces, necessary visual feedback from the microrobot should be supplied to an operator. The precise tracking of microrobots remains a fundamental challenge in medical applications due to the limited visibility and constrained working spaces within the human body. Various tracking methodologies for microrobots have been proposed, such as magnetic localization using Hall-effect sensor arrays [5,6,7,8], optoacoustic imaging [9,10], and image processing via deep learning-based approaches [11,12,13]. Yang et al. developed a microrobot tracking system capable of simultaneously tracking three magnetic objects via a magnetic sensor array using a hybrid optimization strategy combining Particle Swarm Optimization and Levenberg–Marquardt methods [14]. Zheng et al. presented an approach for automatic 3D navigation control of untethered magnetic microrobots in centimeter-scale workspaces [15]. Overall, at short distances, magnetic tracking offers advantages in terms of simplicity, cost and ability to sense through opaque materials.

Microrobot tracking during medical operations remains a challenging task, as the regions where microrobot operations occur are non-trivial to observe from outside the workspace [16]. Hong et al. presented a real-time holographic tracking system that integrates digital holography with 5-DoF electromagnetic manipulation for microrobots, enabling three-dimensional position tracking [17]. Li et al. developed a system for automated in vivo navigation of magnetically driven microrobots using an optical coherence tomography (OCT) imaging system, which provides high-resolution, real-time tracking in various fluid environments [18]. Martel et al. demonstrated a methodology for the automatic navigation of an untethered magnetic device within the arterial system of a living animal by utilizing a standard MRI system to simultaneously perform localization and propulsion [19]. Lee et al. introduced an on–off actuation strategy for simultaneous microrobot control and localization. Control is achieved through position-independent magnetic force, while localization utilizes a non-vision-based approach with Hall-effect sensors [20].

Hybrid tracking systems that integrate other technologies alongside magnetic localization overcome the limitations of singular methodologies by combining multiple sensing modalities. Mori et al. presented a hybrid bronchoscopy tracking system that combined electromagnetic sensor tracking with intensity-based image registration to achieve high-precision navigation within the bronchial tree [21]. Similarly, Nakamoto et al. introduced a magneto-optic hybrid tracking approach to dynamically correct magnetic field distortions in electromagnetic tracking systems used for laparoscopic 3D ultrasound navigation [22]. In most surgical navigation applications, optical tracking is frequently utilized for its advantages, such as large workspaces, high precision, and low cost. However, tracking an object such as an untethered microrobot within a closed environment, like the human body, requires more sophisticated alternative tracking approaches. Each tracking method has its own advantages and disadvantages, such as cost, robustness, complexity, tracking distance, and workspace constraints. Hence, hybrid tracking systems utilizing two or more methodologies are developed to address the limitations of individual approaches.

As shown in Table 1, the proposed method estimates 4-DoF pose (2 translation and 2 rotation DoFs) with an RMS error of 0.498 mm using four physical Hall-effect sensors. Each physical Hall-effect sensor provides readings at multiple known positions via a single DoF sweeping motion provided by the integrated sensor apparatus servo actuator, combined with its optical tracking. Therefore, the proposed method extracts more information per physical sensor compared with fixed sensor arrays. However, the time delay caused by the sweeping motion affects the tracking period performance of the system.

Magnetic localization using Hall-effect sensor arrays is low-cost and can sense through biological tissue and other opaque materials, but it has a limited sensing depth and workspace [5,6,8,20] and often requires dense sensor arrays to achieve high accuracy [5,8]. Optoacoustic imaging provides high resolution but requires specialized equipment and is limited by light attenuation in tissue [9,10]. Optical tracking is highly accurate over large workspaces but requires a direct line of sight and cannot track objects inside occluded regions such as the human body [5,23]. MRI-based localization enables simultaneous propulsion and tracking but places strict constraints on the materials used [19].

In light of the current literature, this study proposes a hybrid microrobot tracking system that utilizes optical and magnetic sensors to accurately track the pose of an untethered microrobot within an environment where traditional methods of surgical feedback are ineffective. The proposed system consists of a manufactured Hall-effect sensor apparatus with a single-DoF planar sweeping capability, low-cost commercial optical tracking cameras, and an industrial robot manipulator arm. The real-time tracking of the sensor apparatus is performed by cost-effective optical tracking cameras. A point-based registration methodology [24,25] is utilized to calculate the relationship between the coordinate frames of the robot manipulator [23] and the sensor apparatus. The sensor apparatus is calibrated via an artificial neural network (ANN) approach. The real-time pose of the microrobot is estimated by applying the developed sensor data aggregation and fusion method to data collected from the calibrated sensor apparatus and optical tracking cameras.

## 2. Methodology

### 2.1. Hardware and Software

One of the main goals of this study is to accurately track the pose of an untethered microrobot within an environment where traditional surgical feedback methods are ineffective. The hardware necessary to perform effective tracking under the mentioned conditions includes but is not limited to motion tracking cameras, infra-reflective markers, precisely manufactured stylus tool(s), and robot manipulator(s). Utilizing this hardware, the pose of any point, vector, or rigid body within a tracked volume can be measured within the view of the motion capture cameras, provided that their calibration is precisely carried out alongside the registration of the stylus tool and robot manipulator. These calibration and registration procedures enable tracking in a workspace where passive infra-reflective markers are visible to the motion capture cameras.

In order to develop a system capable of precise and accurate microrobot tracking, the use of motion capture cameras alone is not sufficient, as the microrobot operates within an enclosed workspace. To address this, a sensor apparatus was designed to calculate the 3D position and orientation of the microrobot using Hall-effect sensor data and an artificial neural network approach within the patient workspace. The utilized hardware and their respective calibration/registration methods are provided in Table 2.

**Figure 1:**
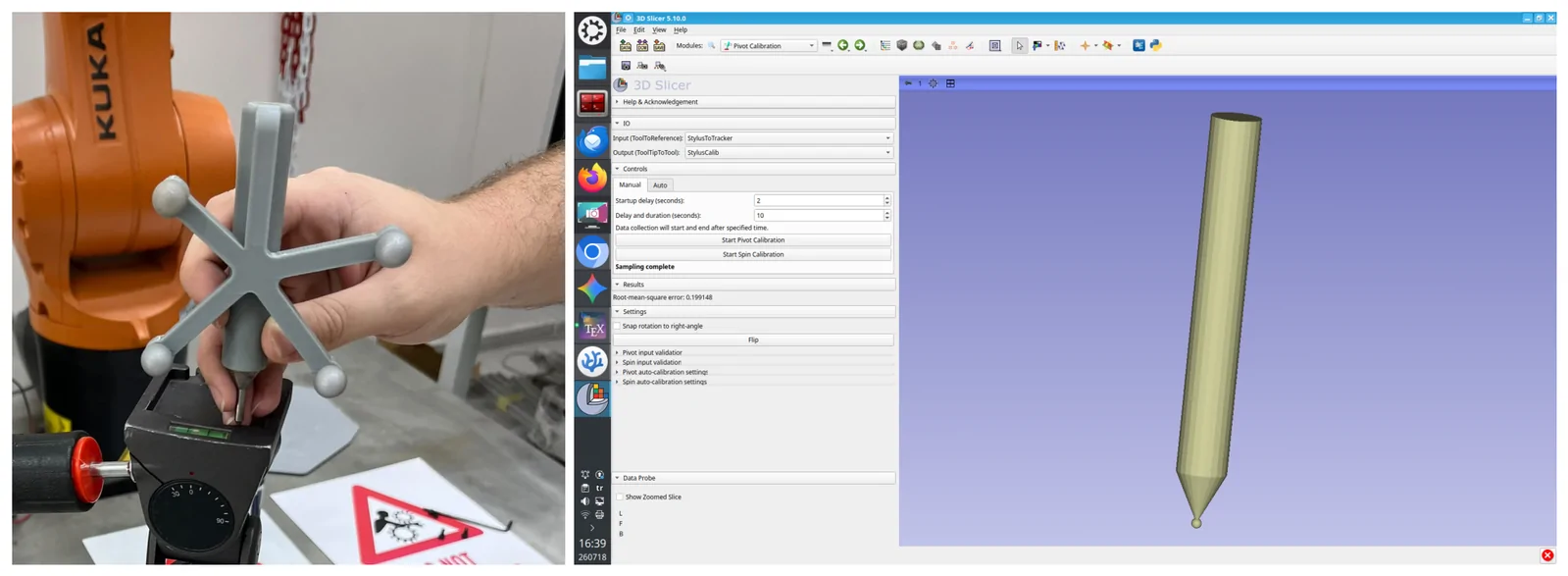
Utilized stylus tool with four fixed infra-reflective markers and virtual representation inside 3D Slicer.

**Figure 2:**
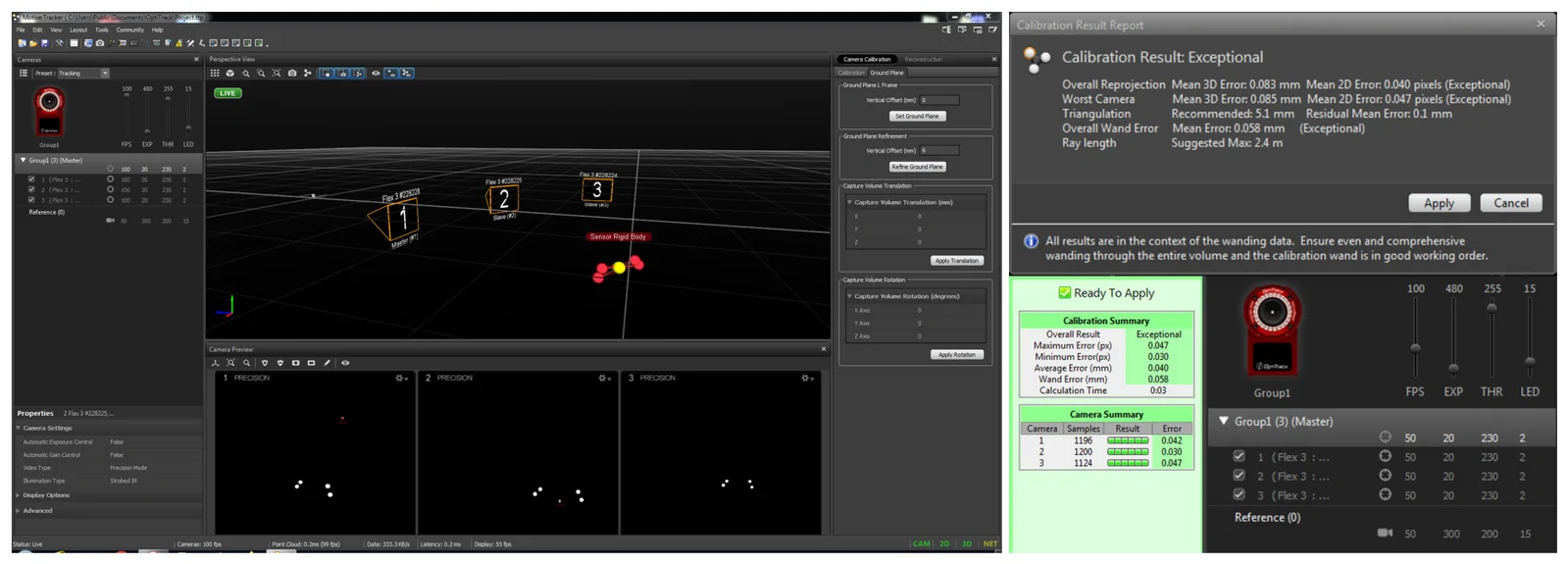
Motive Tracker software and calibration results screen.

Three cost-effective Optitrack Flex 3 infrared cameras are used as the motion capture system in this study. These cameras can be calibrated using Motive Tracker, the official software provided by Optitrack. The calibration procedure requires moving a special tool equipped with three passive infra-reflective markers within the desired workspace volume. A stylus tool with fixed infra-reflective markers is shown in Figure 1. The 2D projected positions of these markers on the camera sensors are sampled for each motion capture camera to calculate the 3D relations between the cameras and the virtual workspace within the Motive Tracker software program, as shown in Figure 2.

Following this procedure, the calibrated motion capture cameras are able to perform the real-time position and orientation tracking of infra-reflective markers and rigid bodies with at least three attached markers. The stylus tool and sensor apparatus are tracked within the workspace by constructing virtual rigid bodies using infra-reflective markers. The stylus tool is calibrated using the spin and pivot calibration methodologies provided by the SlicerIGT extension module of 3D Slicer software. To properly track other rigid bodies via the setup, a rigid body registration methodology must be applied to calculate the spatial transformations between the virtual rigid bodies constructed by the motion capture cameras and the coordinate frames of the tracked objects.

**Figure 3:**
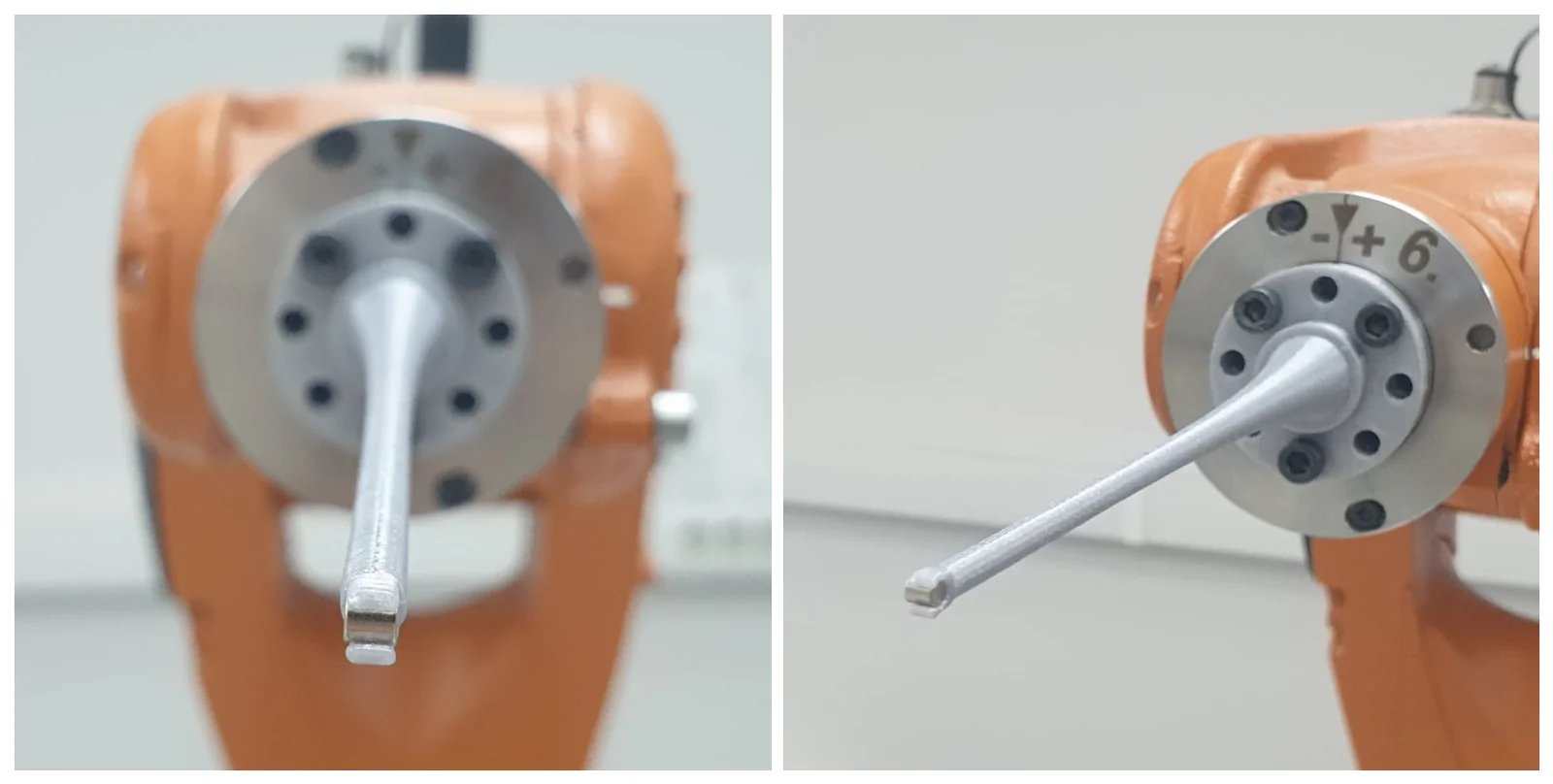
Microrobot holder part attached to end effector of the robot manipulator.

For the purpose of mimicking untethered motion of a microrobot inside the workspace, an industrial KUKA KR6 r900 sixx robot manipulator (KUKA, Augsburg, Germany) is integrated into the system after the relation between its base frame and global frame has been identified via the robot registration methodology [23]. The microrobot carried by the end effector of the manipulator is shown in Figure 3.

**Figure 4:**
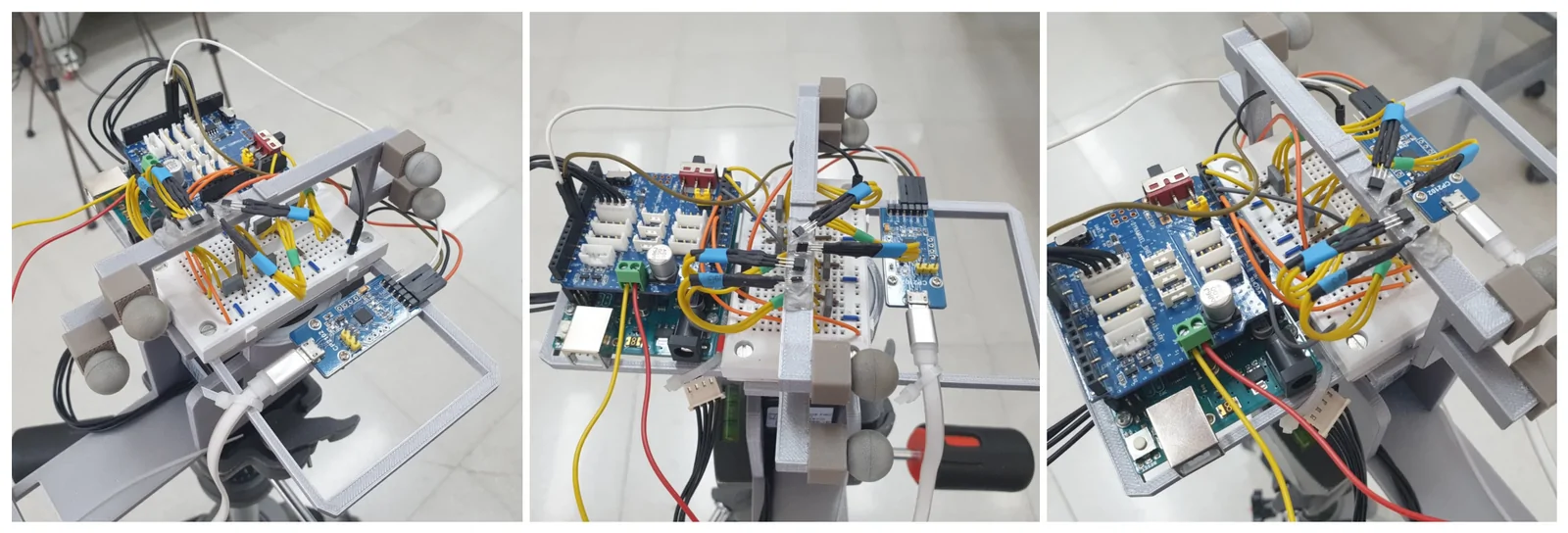
Constructed sensor apparatus containing four Hall-effect sensors and four fixed infra-reflective markers.

The sensor apparatus consists of four Asahi Kasei Microdevices EQ730L Hall-effect sensors (Asahi Kasei Microdevices (AKM), Tokyo, Japan) for measuring the magnetic field formed by the microrobot; a single Dynamixel XM430 smart servo motor (ROBOTIS Co., Ltd., Seoul, Republic of Korea) to enable single-DoF planar sweeping motion for scanning; an Arduino UNO microprocessor board equipped with a Dynamixel shield extension for data acquisition, serial communication, and servo control; and a TTY-to-USB converter for communication. The manufactured sensor apparatus is shown in Figure 4. On the computer side, Robot Operating System 2 (ROS2) software is utilized for data synchronization between the optical tracking system and the sensor apparatus, as well as for controlling the overall system. Data acquisition is performed from two sources, the optical tracking system and the sensor apparatus. Synchronization between these data sources is critical to deriving a meaningful relationship between the data to establish an effective calibration methodology for the sensor card. In order to simulate a magnetically driven microrobot, a robot manipulator is utilized. In this scenario, the sensor apparatus base is fixed, and the microrobot is moved to the desired pose by a holder attached to the end effector of the robot manipulator.

### 2.2. Registration and Pose Estimation Methodology

The developed hybrid tracking system aims to achieve low fiducial registration error (FRE) and target registration error (TRE) [28] in microrobot location identification, even in regions of the human body where optical or visual tracking alone is not feasible. To achieve this goal,

- Accurate calibration of the motion tracking cameras placed around the workspace is performed.
- Precise registration of the industrial KUKA KR6 sixx r900 robot arm (robot manipulator) within the workspace is performed via calibrated motion capture cameras to relate their respective reference frames.
- The relationship between the sensor apparatus frame and the global base frame is described.
- The pose of the microrobot is calculated by utilizing Hall sensor data captured by the sensor apparatus.

This procedure combines the benefits of optical tracking, such as low fiducial registration error and target registration error, with the advantages of magnetic tracking methodologies. The proposed combination will enable surgical navigation in areas where obtaining visual feedback is not possible or efficient.

Calibration procedures for each related frame are performed in order to track the position of the microrobot. These procedures are environment registration for the robot manipulator *K* frame, motion capture system calibration for the *M* frame, and pivot/spin calibration for the stylus tip St frame. By establishing the relationships between these frames, any point in the optical tracking workspace can be measured by the stylus tool and reached by the robot manipulator. After the registration procedures are successfully completed, a calibration methodology for the sensor apparatus is also performed.

**Virtual Environment:** Once the relationships between the existing frames are calculated, a virtual environment containing virtual representations of the sensor apparatus, robot manipulator end effector, microrobot, and stylus tool is created using 3D Slicer software.

**Figure 5:**
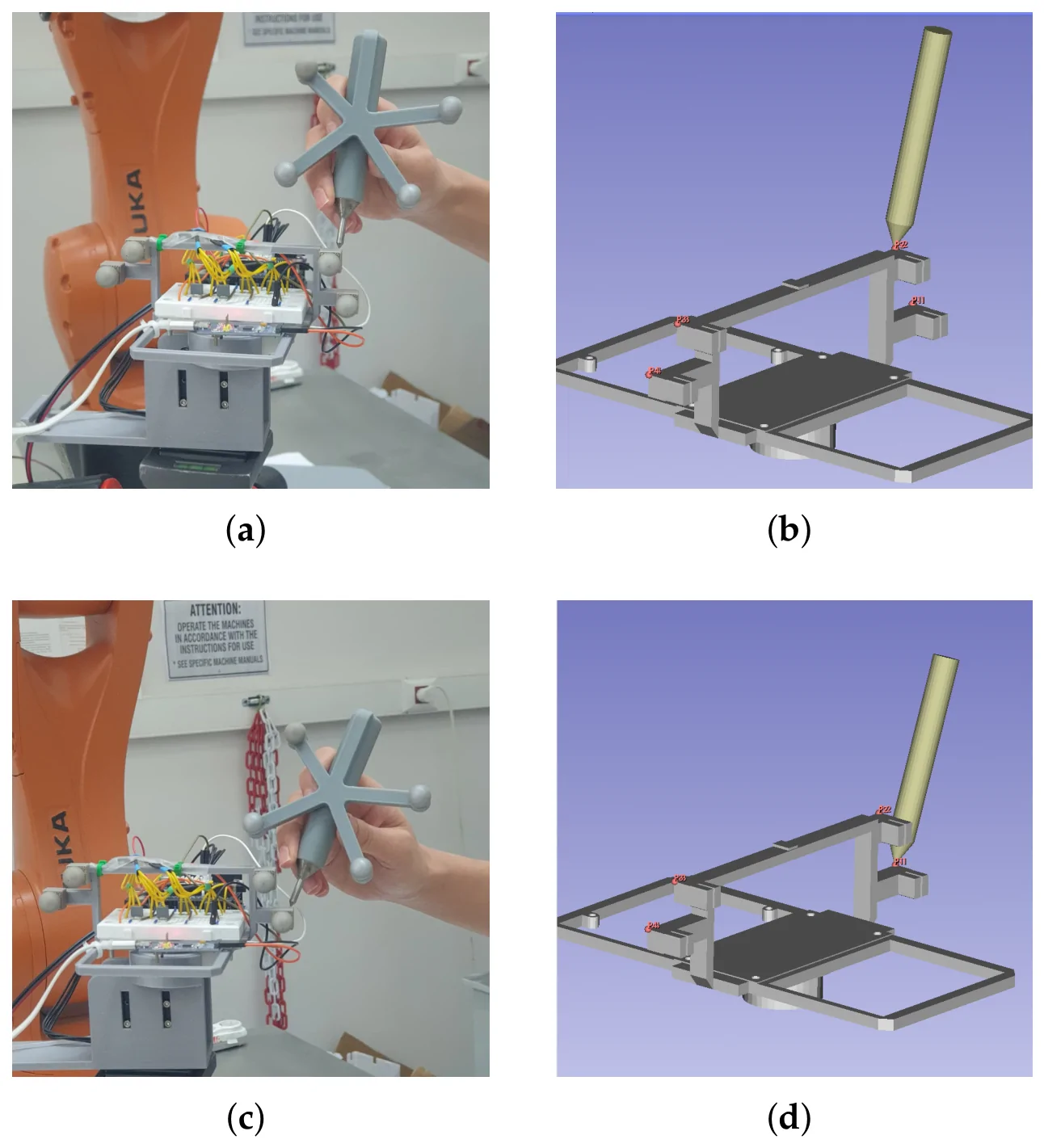
Point data collection via a calibrated stylus tool. (**a**,**c**) Stylus tool tip placed on a fiducial point of the sensor apparatus. (**b**,**d**) Stylus tool and sensor apparatus as seen in the 3D Slicer virtual environment during fiducial point data collection.

**Stylus Tool:** Using a calibrated stylus tool, any point in real space can be mapped to virtual space. The stylus tool, shown in Figure 1, is tracked within the workspace with the help of a virtual rigid body (*I*) constructed with Motive Tracker from four infra-reflective markers fixed to the tool. The stylus tool is calibrated using spin and pivot calibration methodologies. The point data collection procedure within the constructed virtual environment in 3D Slicer software is shown in Figure 5.

Spin and pivot calibration is utilized to calculate the ${\hat{z}}_{U}$ axis of the pointer tip point reference (*U*) with respect to the pointer tool reference (*I*), and they help determine the tip point position of the stylus tool with respect to the stylus tool reference. This point represents the origin of the stylus tip point reference δU  I, and is the most important parameter for performing measurements in the relevant workspace via the stylus tool. Spin calibration is performed by fixing the position of the tip point of the stylus tool and gently spinning it around its spin axis. Assuming that the spin axis and the ${\hat{z}}_{U}$ axis of the stylus tip point reference are coincident, the R  UI rotation matrix between the stylus tool reference and the stylus tip point reference can be calculated numerically. Spin calibration ensures that the orientation of the stylus tool is consistent between the real and virtual environments. During pivot calibration, the position of the stylus tip point is fixed, and the stylus tool is moved like the platform of a spherical mechanism. Samples collected during this motion are then utilized in the pivot calibration calculation. The R  UI rotation matrix found in spin calibration and the δU  I vector calculated during pivot calibration can then be utilized to construct the T  UI transformation matrix. Both spin and pivot calibration procedures are performed utilizing 3D Slicer software with the SlicerIGT extension module [23].

**Rigid Body Registration**: The point-based registration methodology is utilized to find the relationship between an object assumed to be a rigid body and its virtual representation. Distinctive and unobscured points selected from the surface of the rigid body can be defined as fiducial points. The core strategy for mapping two reference frames (*A* and *B*) to each other involves utilizing registration methodologies with position data obtained from *n* fiducial points using a stylus tool. Following the registration procedure, the FRE is calculated as the root mean square of the spatial distance between each pair of real and virtual fiducial points ($e_{i}\;\;i=1,2,\ldots ,n$).

(1) [ρi  A1]=T  BAρi  B1+ei0i=1,2,…,n

In Equation (1), ρ   A=ρx  Aρy  Aρz  AT and ρ   B=ρx  Bρy  Bρz  BT are position vectors defining the corresponding points with respect to the *A* and *B* frames. The T  BA transformation matrix includes a rotation matrix R  BA=x^B  Ay^B  Az^B  A that defines the relative orientation between the references, and a translation vector ρB  A=ρBx  AρBy  AρBz  AT that defines the relative distance vector between the origins of the references (Equation (2)).

(2) T  BA=R  BAρB  A0001

**Figure 6:**
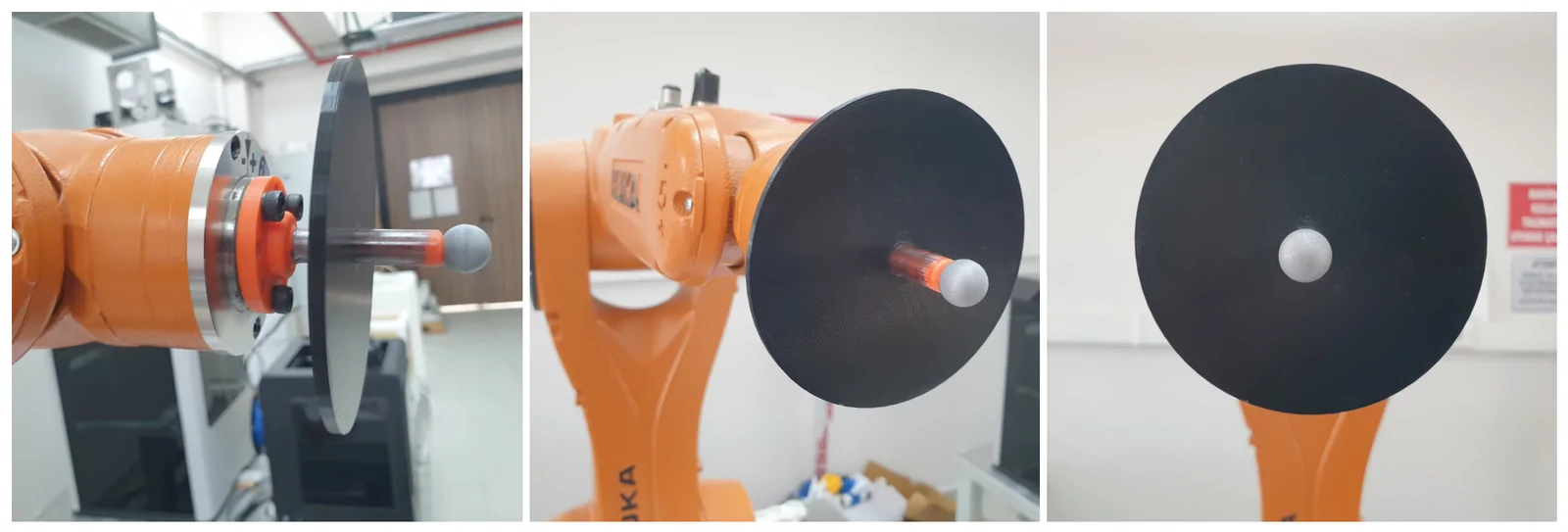
A 3D-printed end effector part with a single infra-reflective marker.

**Robot Manipulator Registration:** For the successful utilization of the 6-DoF KUKA KR6 R900 SIXX serial robot manipulator, the T  RK transformation matrix relating the manipulator frame (*K*) and the optical motion capture system frame (*R*) is calculated. Similar to the previously discussed rigid body registration procedure, robot manipulator registration requires point position sets described with respect to both frames, ρPi  K and ρPi  Ri=1,2,3,…,n. To carry out the registration procedure, a part with a single infra-reflective marker is attached to the robot manipulator’s end effector to collect the point sets (Figure 6).

A 6 × 6 × 6 three-dimensional grid of points, totaling 216 points, is fitted inside a cuboid volume. Required position sets are constructed by moving the end effector tip to point-to-point positions with arbitrary orientations relative to the robot reference frame, and measuring these positions using an attached marker with motion capture cameras relative to the optical motion capture system reference frame. The order of point arrival by the robot end effector is randomized during point collection. Furthermore, the point position sets are assigned a set of Euler angles for each point as $\left[\begin{array}{ccc} \pm \frac{\pi }{36} & \pm \frac{\pi }{36} & \pm \frac{\pi }{36} \end{array}\right]$ radians. After constructing the point sets, robot registration using the drift compensation methodology detailed in [23] is performed.

**Figure 7:**
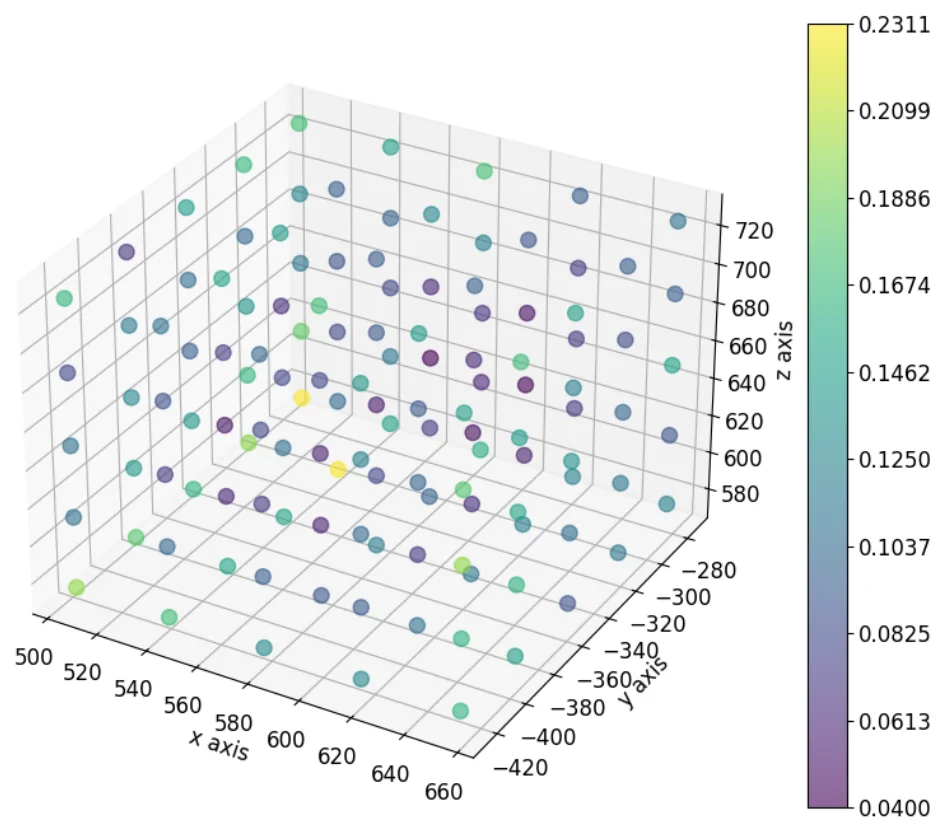
Heatmap of error at points of robot manipulator workspace.

Errors ($e_{i}$) are calculated utilizing Equation (1). Gradient of errors (mm) at each point is calculated and shown in Figure 7. Root mean square (RMS) of the robot registration error is found to be 0.121 mm.

### 2.3. Magnetic Tracking of Microrobot via Sensor Apparatus

After the successful application of registration procedures, the poses of the robot manipulator and sensor apparatus are known in real time. Thus, every transformation, except the transformation between the sensor apparatus and the microrobot, can be calculated relative to a reference frame to construct the hybrid tracking system in a virtual environment.

(3) T  M0T  SrMT  SSrT  RS=T  R0

At this point, the transformation between the sensor apparatus frame and the microrobot (T  RS) is calculated to close the transformation chain between the reference frame and the microrobot (Equation (3)). In the transformation chain shown in Equation (3), the transformation between 0 (**reference frame**) and *M* (**motion capture frame**) (T  M0) is constant and arbitrary, the transformation between *M* and $S_{r}$ (**sensor apparatus virtual rigid body frame**) (T  SrM) is tracked in real time by the motion capture system, the transformation between $S_{r}$ and *S* (**sensor apparatus frame**) (T  SSr) is constant and calculated from the rigid body registration methodology, and lastly, the transformation between *S* and *R* (**microrobot frame**) (T  RS) is calculated in real time for the tracking of the microrobot.

In order to construct the T  RS transformation in real time, an ANN model is trained using a dataset collected with the aid of the robot manipulator. The data collection procedure below is followed:

- Determine the data collection volume with respect to the *S* sensor apparatus frame, and divide it into a grid of points.
- Using the robot manipulator, move the microrobot to a point on the grid with a known pitch orientation. Note that although only the pitch angle of the microrobot is manipulated, the yaw angle changes relative to the virtual scanning plane depending on the microrobot’s position within the data collection volume.
- Collect Hall-effect sensor data while the microrobot is held at the target point.
- Move the microrobot to another point on the grid and repeat the data collection process until all points on the grid are covered.

**Figure 8:**
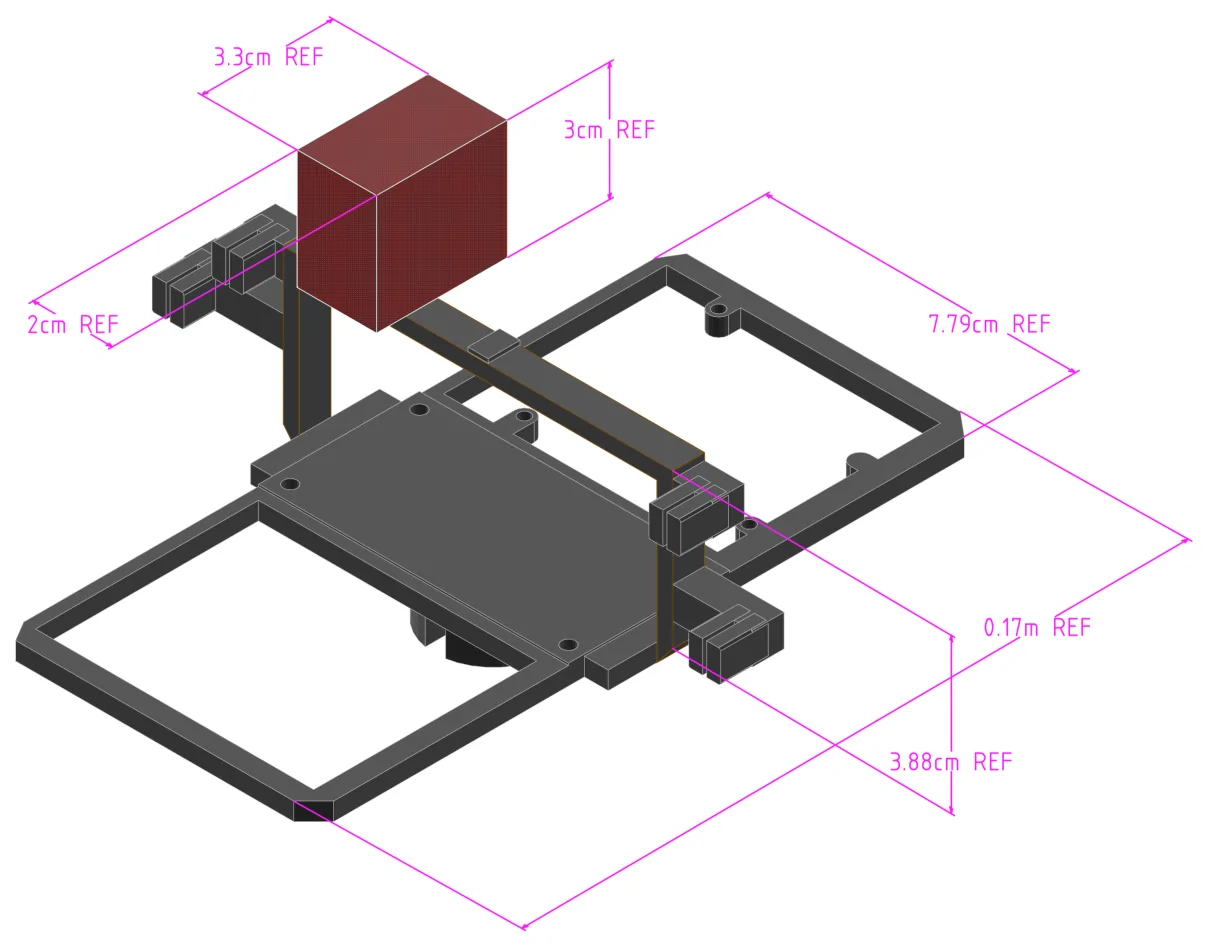
The data collection volume.

In this study, a 3 mm by 3 mm cylindrical N35 NdFeB magnet is used as the microrobot. The data collection workspace was defined as a three-dimensional grid of target points, as shown in Figure 8. The grid path was augmented with randomized pitch angles for each target point. On the sensor apparatus, four EQ730L Hall-effect sensors were placed 6 mm apart with arbitrary orientations, starting from a 15 mm distance from the rotation axis of the servo motor.

Note that the microrobot must be placed at a correct point by the robot manipulator. For this task, the transformation chain given in Equation (5) is utilized.

(4) T  SrMT  SSr=T  SM

(5) T  K0T  MKT  SM=T  S0

In Equation (5), the T  SM transformation is known and can be calculated in real time from Equation (4). The transformation between *K* (**robot manipulator frame**) and *M* (**motion capture frame**) (T  MK) is constant and calculated via a robot registration procedure [23]. The T  K0 transformation between 0 (**reference frame**) and *K* (**robot manipulator frame**) is assumed to be the identity. Consequently, the transformation T  S0 is known and calculated in real time; as a result, the robot manipulator end effector can be moved to any position and orientation with respect to the *S* sensor apparatus frame, including any point within the data collection volume.

In order to train the ANN, a comprehensive dataset mapping Hall-effect sensor readings to precise spatial poses was generated using an automated data collection program implemented in ROS2. This program provides synchronization and communication with the KUKA KR6 R900 SIXX serial robot manipulator via the KukaVarProxy protocol and the sensor apparatus via a serial interface. It also collects real-time motion capture data from Motive software via the NatNet protocol and streams the relevant transforms to 3D Slicer via the OpenIGTLink protocol for real-time visualization. The procedure of the data collection program can be summarized as follows:

- The program selects a target pose from the pre-generated grid.
- The robot manipulator carries the microrobot to the target pose.
- To ensure spatial accuracy, the system continuously monitors the manipulator’s current pose via feedback. Data collection only commences once the robot is verified to be within a predefined tolerance (±0.2 mm and ±2.0°).
- Once the target is reached, the sensor apparatus performs several sweep motions using the integrated Dynamixel servo motor.
- The system logs a vector containing the grid point index, the robot’s Cartesian pose, raw magnetic field readings, and the current pose of the sensor apparatus as tracked by the optical system.

**Figure 9:**
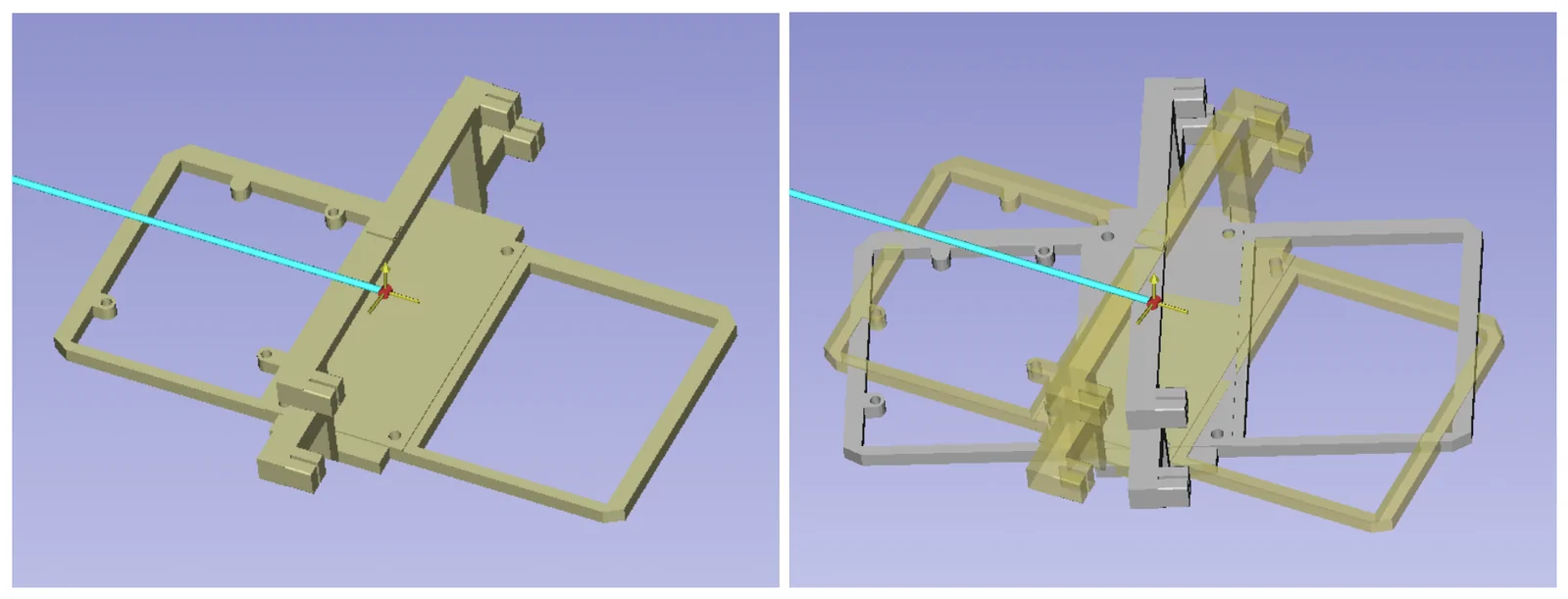
Sensor apparatus sweeping motion with respect to a set reference degree in 3D Slicer software, monitored in real time.

As illustrated in Figure 9, the sensor apparatus collects data while rotating within the interval of −22.5° and 22.5° with respect to a set reference angle, performing a radar-like sweep motion with the help of an XM430 Dynamixel Smart Servo while recording the readings from the Hall-effect sensors. This design was adopted to minimize Hall-effect sensor usage while adding another DoF to the robot manipulator end effector for easier motion planning. Overall, this data collection method provides sets of Hall-effect sensor data sampled along semi-circular arcs that are coincident with the motion axis of the servo motor.

#### 2.3.1. Sensor Data Pre-Processing and Fusion

Once the optical tracking data for the sensor apparatus are determined from Equation (3) and the Hall-effect sensor data are collected, the raw data must be cleaned and pre-processed to create a training dataset for the ANN model. Therefore, a Python-based data pre-processing program was developed. Initially, the program takes a list of Hall-effect sensor readings ($s_{i}$) for i=0,1,2,…,n, together with the corresponding Ti S0 transformation matrices. The steps of the pre-processing procedure are described below:

- Process all Ti S0 to extract and record the rotation angle ($\theta_{i,i+1}$) between consecutive steps using rotation matrix-to-axis–angle conversion.
- Determine the points where $\theta_{i,i+1}$ changes direction, and split the sensor data readings into ranges between these points.
- Define the number of virtual sensors *j*, and place them within these ranges using either equal, Chebyshev–Labatto, or half-cosine spacing, depending on the virtual scanning plane placement.
- Determine the values of these virtual sensors by calculating a weighted mean using squared weights from the real sensor readings.

The training dataset consists of two parts: the input data (X), which contain the parameters fed into the ANN model, and the corresponding output data (y), which include the parameters predicted by the model. For the ANN input training dataset, sensor readings must be sampled at exact intervals to ensure meaningful output from the model. By utilizing virtual sensor positions calculated from real sensor readings, synchronization between the servo motor position and the Hall-effect sensor readings is no longer necessary. Furthermore, a virtual scanning plane is defined normal to the sweeping motion plane at a known angle. Since the sensor apparatus is tracked in real time via optical motion capture, the exact spatial pose of this virtual plane is known. By assuming that the microrobot resides on this virtual plane during a measurement window, the translational search space is reduced to 2D space.

Next, to optimize the convergence of the ANN, the 6-DoF pose of the microrobot relative to the sensor apparatus frame (T  RS) was reduced to a 4-DoF pose. This reduction is justified by the following constraints:

- The ANN model is expected to estimate the T  RS transformation matrix. However, since the training data are collected from a virtual plane, the microrobot is assumed to translate freely on this plane. Thus, the translation vector can be defined as ρR  S=0ρRy  SρRz  ST.
- The microrobot is a cylindrical permanent magnet. Because its magnetic field is invariant to rotations about its own longitudinal axis, the yaw orientation can be ignored.

The reduced transformation matrix is given in Equation (6).

(6) T  RS=0Rα RSRβ RSρRy  SρRz  S0001=cosβ0sinβ0sinαsinβcosα−sinαcosβty−cosαsinβsinαcosαcosβtz0001

The T  RS transformation matrix can be constructed using four parameters: the rotation angles α and β and the translation distances $t_{y}$ and $t_{z}$. When constructing the output data, these parameters are encoded as follows:

(7) $$\begin{array}{c} t_{y}=y_{2}\sqrt{1-y_{1}^{2}}\\ t_{z}=y_{2}\;y_{1}\\ \alpha =\arcsin y_{3}\\ \beta =\arcsin y_{4} \end{array}\;\;and\;\;\begin{array}{c} y_{1}=\cos\theta \\ y_{2}=r\\ y_{3}=\sin\alpha \\ y_{4}=\sin\beta \end{array}\;\;where;\;\;\begin{array}{c} 0\leq \theta \leq \pi \\ \frac{-\pi }{2}\leq \alpha \leq \frac{\pi }{2}\\ \frac{-\pi }{2}\leq \beta \leq \frac{\pi }{2} \end{array}$$

**Figure 10:**
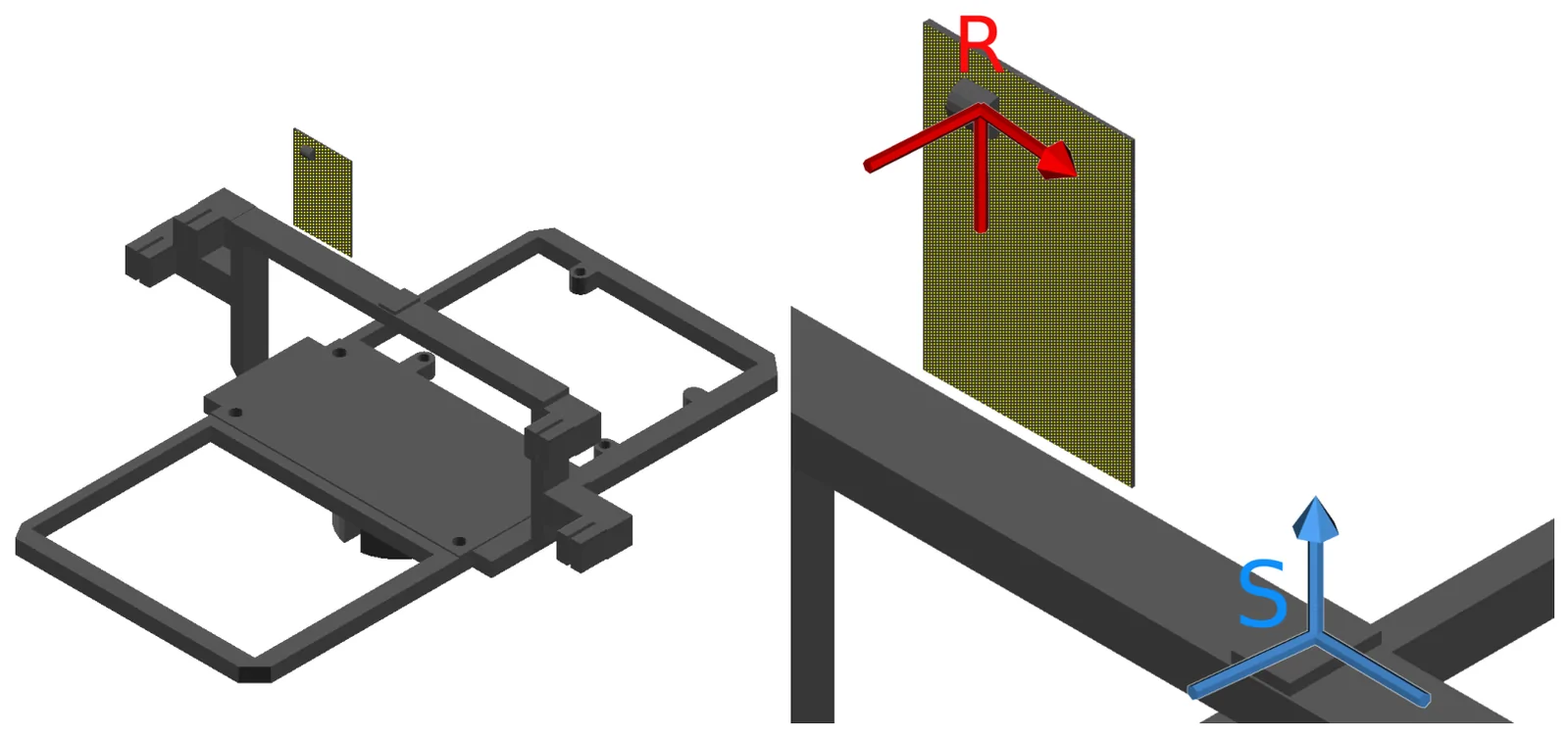
*S* sensor apparatus frame and *R* microrobot frame.

The output data (y) of the ANN model ($y_{1}$ to $y_{4}$) are defined as shown in Equation (7). These values are selected to best fit the frame placements shown in Figure 10, although there is a manageable ambiguity regarding the pole placement of the microrobot. By inserting the ANN output parameters into Equation (6), the T  RS transformation matrix can be calculated from the model’s predictions (Equation (8)).

(8) T  RS=1−y420y40y3y41−y32−y31−y42y21−y12−y41−y32y31−y321−y42y2y10001

#### 2.3.2. Training ANN Models

Using the Scikit-Learn library, the datasets (X, y) are split into training (75%), test (15%), and validation (10%) subsets to train two distinct ANN models. The input data X are scaled using the Yeo–Johnson power transformer implementation from Scikit-Learn. In contrast, the output data y remain unscaled, as these are trigonometric values, with the exception of $y_{2}=r$, which is scaled using a MinMax scaler from −1 to 1. For *n* virtual sensors, the ANN models are designed as fully connected multilayer perceptrons. The layer dimensions are n,512,256,128,2 for position prediction ($y_{1},y_{2}$) and n,128,64,32,2 for orientation prediction ($y_{3},y_{4}$). Additionally, the Adam (Adaptive Moment Estimation) optimizer and the ReLU (Rectified Linear Unit) activation function from Scikit-Learn are utilized during training.

**Figure 11:**
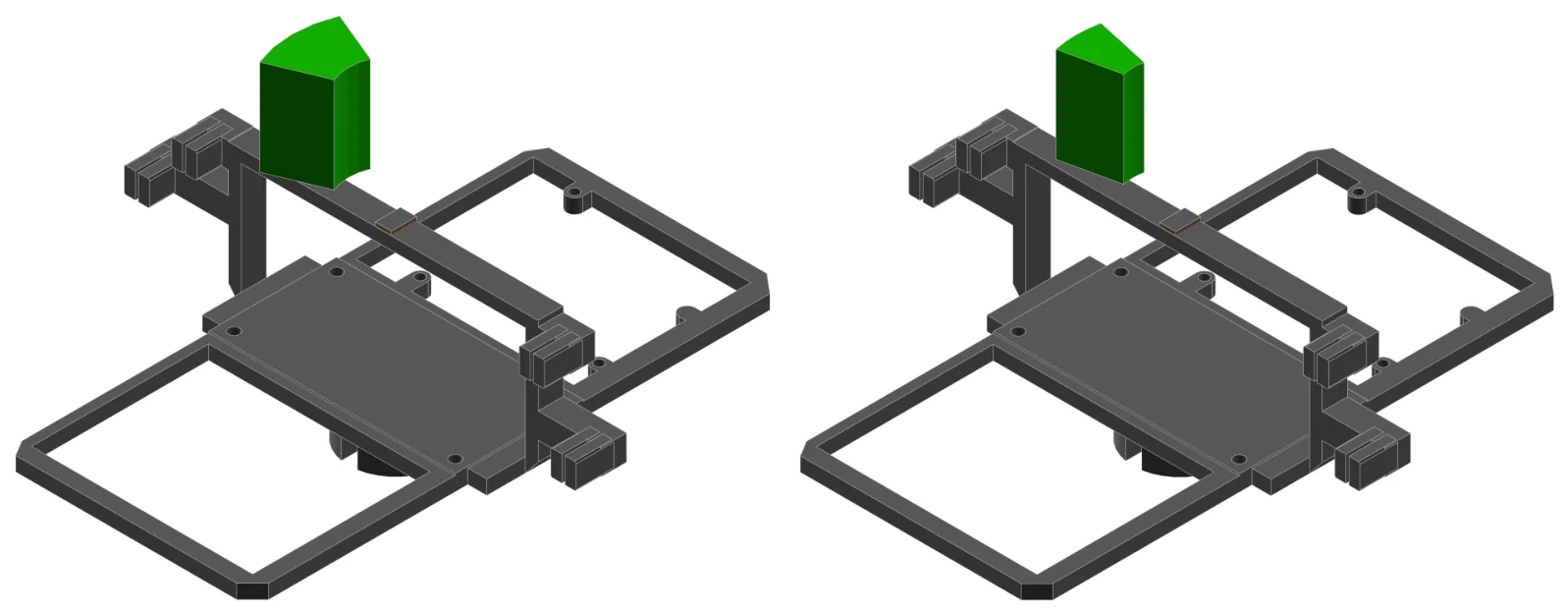
Maximum (45°) and minimum (25°) usable 3D scanning volume of the sensor apparatus.

Virtual sensors can be placed arbitrarily along the arcs of the sensor data generated during the sweep motion of the sensor apparatus. If the arc used by the virtual sensors is smaller than the actual arc traversed by the apparatus, a 3D scanning volume can be created, as shown in Figure 11.

This volume is created by taking multiple slices, achieved by offsetting the virtual sensors within a single sweep of real sensor data. The size of this scanning volume depends on the ratio between the arc angle of the virtual sensors and the arc angle of the actual sweep motion of the sensor apparatus. The ANN model outputs at each virtual sensor offset are then compared with each other and with previous tracking data to determine the most likely pose of the microrobot. For an actual sweep arc of 45°, the achievable 3D scanning volumes are listed in Table 3.

## 3. Results and Hardware Verification

**Figure 12:**
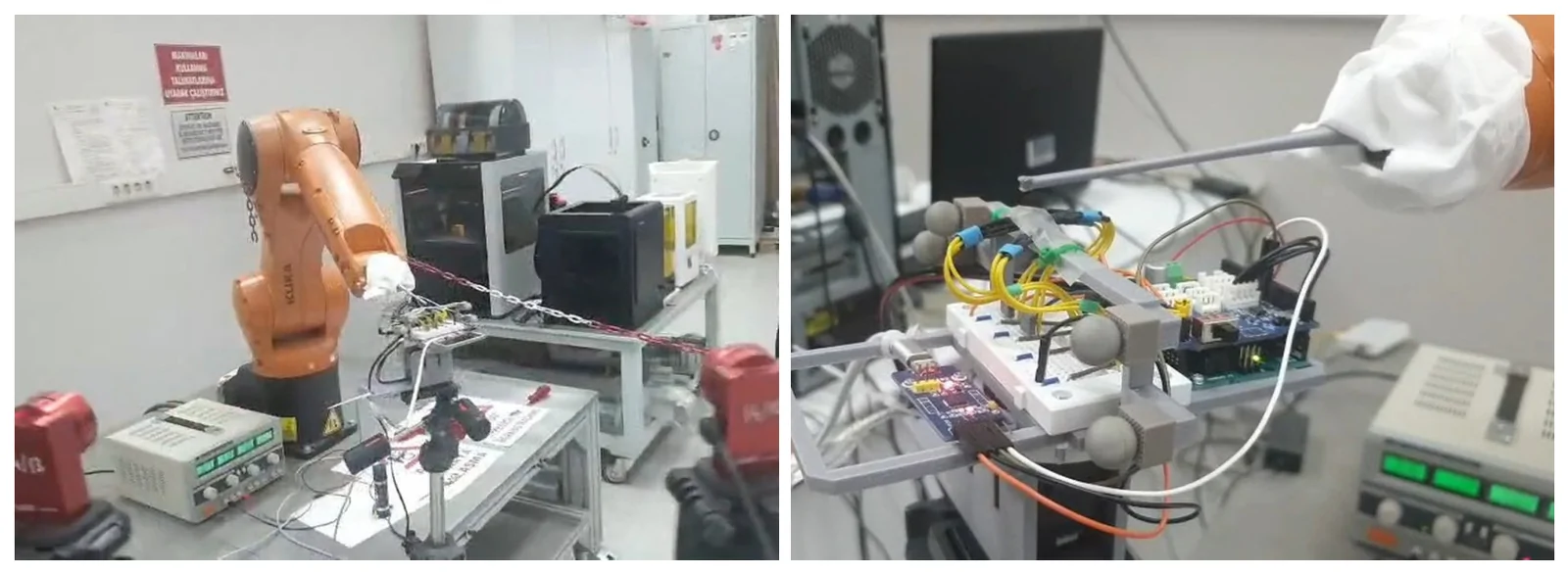
Hardware verification setup containing calibrated/registered robot manipulator, motion capture cameras, sensor apparatus and microrobot.

The constructed hardware verification setup is shown in Figure 12. The hardware component of the setup consists of a 3 mm by 3 mm cylindrical N35-grade NdFeB magnet as the microrobot, the manufactured sensor apparatus for the real-time tracking of the microrobot, a KUKA KR6 R900 SIXX robot manipulator to actuate the microrobot during data collection and tracking verification, Optitrack Flex 3 motion capture cameras for the optical tracking of the sensor apparatus, and finally, a stylus tool for performing rigid body registration on the sensor apparatus. On the software side, ROS2 with NatNet, KukaVarProxy, and OpenIGTLink modules were used for data collection, processing, and synchronization between sources; Optitrack Motive was used for optical tracking and calibration; 3D Slicer with the SlicerIGT module was used to create the visualization environment; and Scikit-learn was utilized for ANN model training and inference.

### 3.1. Calibration and Registration Results

Resulting error values for the calibration and registration procedures of each hardware component are provided in Table 4. Sub-millimeter RMS error values were achieved for the optically tracked objects. Note that the sensor apparatus was registered using both a rigid body registration procedure with the stylus tool and optical tracking, and it was calibrated for the magnetic tracking of the microrobot via Hall-effect sensors through a data collection and ANN training procedure.

Using the collected Hall-effect sensor data, ANN models were trained with various node counts, scanning arc measures, and spacing methods. The results for equal spacing and Chebyshev–Labatto spacing are provided in Table 6 and Table 7, respectively.

**Table 5:** Normalized node placement chart for Table 6 and Table 7. Virtual plane placed at 0.0.

	2 Nodes	3 Nodes	4 Nodes	5 Nodes	6 Nodes
Linear spacing	−0.5, 0.5	−0.5, 0.0, 0.5	−0.5, −0.167, 0.167, 0.5	−0.5, −0.25, 0.0, 0.25, 0.5	−0.5, −0.3, −0.1, 0.1, 0.30, 0.5
Chebyshev–Labatto spacing	same as linear spacing	same as linear spacing	−0.5, −0.25, 0.25, 0.5	−0.5, −0.354, 0.0, 0.354, 0.5	−0.5, −0.405, −0.155, 0.155, 0.405, 0.5

**Table 6:** RMS TRE results with equal spacing.

	8°	12°	16°	20°	Row Mean
2 nodes	1.066 mm	0.735 mm	0.930 mm	0.814 mm	0.886 mm
3 nodes	1.075 mm	0.679 mm	0.588 mm	0.798 mm	0.785 mm
4 nodes	0.970 mm	0.749 mm	0.623 mm	0.655 mm	0.749 mm
5 nodes	0.930 mm	0.676 mm	0.544 mm	0.740 mm	0.722 mm
6 nodes	1.075 mm	0.642 mm	0.600 mm	**0.498** mm	**0.704** mm
Column Mean	1.023 mm	0.696 mm	**0.657** mm	0.701 mm	

The lowest values are highlighted in bold.

**Table 7:** RMS TRE results with Chebyshev–Labatto spacing.

	8°	12°	16°	20°	Row Mean
4 nodes	1.124 mm	0.690 mm	0.770 mm	0.657 mm	0.810 mm
5 nodes	1.089 mm	0.719 mm	0.616 mm	**0.510** mm	0.734 mm
6 nodes	1.035 mm	0.596 mm	0.536 mm	0.525 mm	**0.673** mm
Column Mean	1.083 mm	0.669 mm	0.641 mm	**0.564** mm	

The lowest values are highlighted in bold.

The ANN models were also trained with a virtual scanning plane placed at the edge of the scanning arc measure. In this method, the entire 3D scanning volume can be utilized rather than only a portion, as virtual sensors are placed on only one side of the virtual plane, whereas they are placed on both sides when the plane is located in the middle of the scanning arc measure. The results are given in Table 9 for equal spacing and Table 10 for half-cosine spacing.

**Figure 13:**
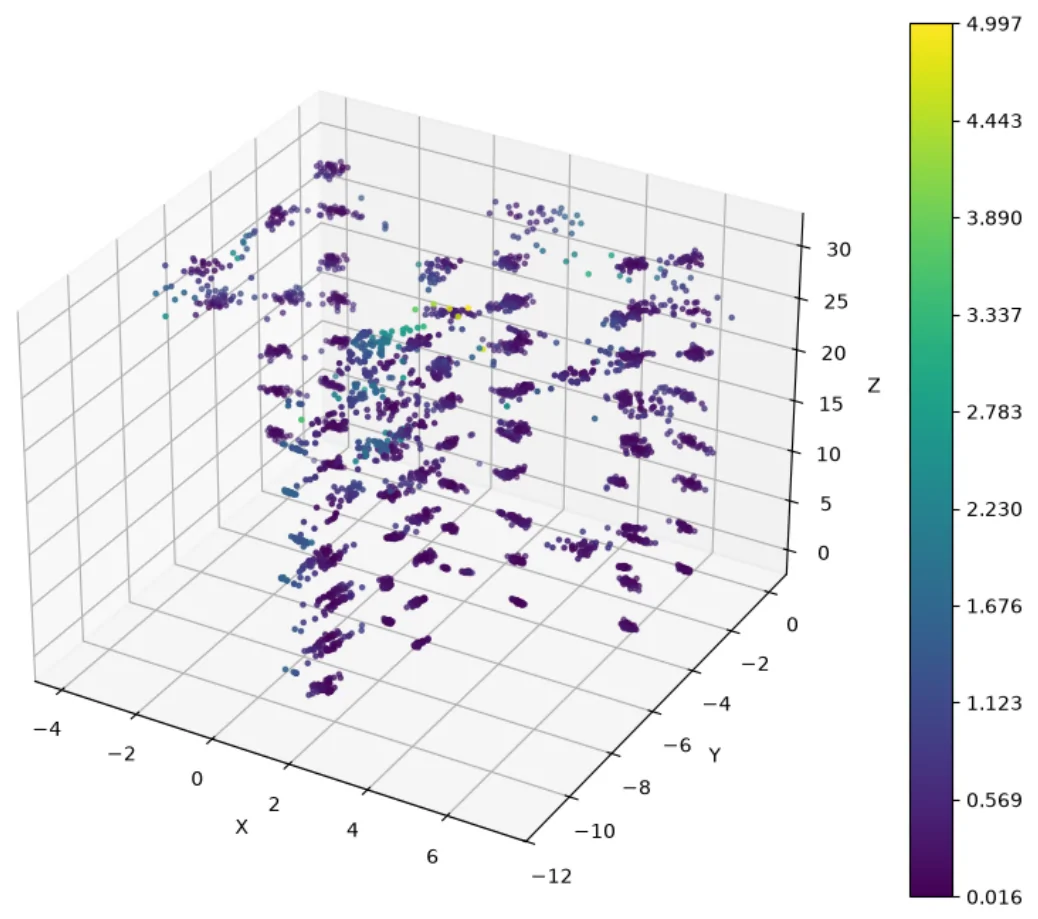
Heatmap of error at microrobot positions with 20° scan arc measure, 6 nodes and equal spacing.

**Figure 14:**
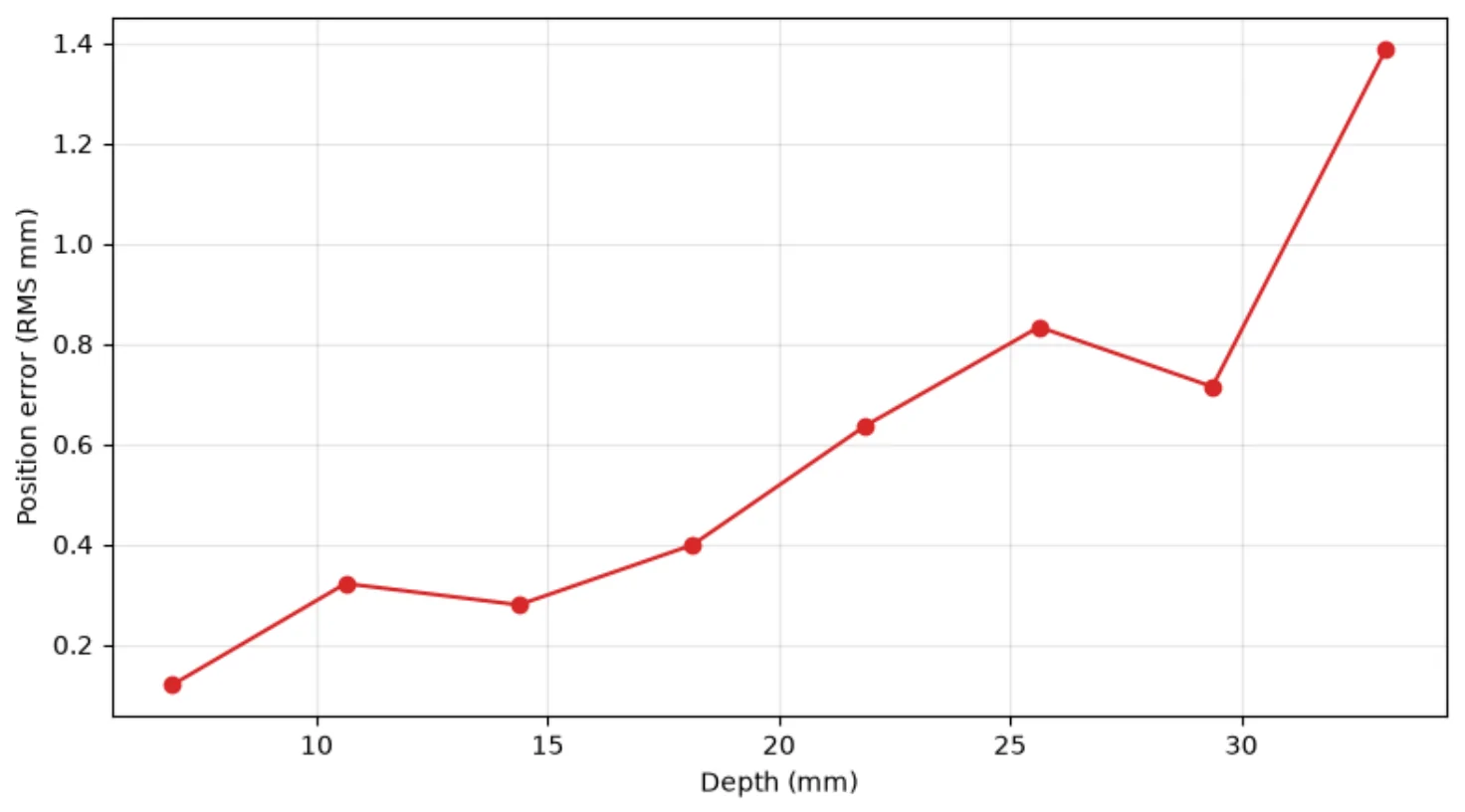
RMS position error versus sensing depth with 20° scan arc measure, 6 nodes and equal spacing.

ANN training was performed with up to six nodes and a 20° arc. Sub-millimeter position error values were achieved for the implemented spacing methods and virtual scanning plane configurations. The error values generally improve with larger scan arc measures and higher node counts. Detailed position error plot for a 20° scan arc measure, six nodes, and equal spacing is shown in Figure 13. Plot of the RMS position error versus sensing depth is shown in Figure 14. With these parameters, the microrobot’s pose was estimated with an RMS target registration error (TRE) of 0.498 mm, an RMS orientation error of 0.014 rad, a position estimation coefficient of determination ($r^{2}$) of 0.979, and an orientation estimation $r^{2}$ of 0.986.

**Table 8:** Normalized node placement chart for Table 9 and Table 10. Virtual plane placed at 0.0.

	2 Nodes	3 Nodes	4 Nodes	5 Nodes	6 Nodes
Linear spacing	0.0, 1.0	0.0, 0.5, 1.0	0.0, 0.334, 0.667, 1.0	0.0, 0.25, 0.50, 0.75, 1.0	0.0, 0.2, 0.4, 0.6, 0.8, 1.0
Half-cosine spacing	same as linear spacing	0.0, 0.293, 1.0	0.0, 0.134, 0.5, 1.0	0.0, 0.076, 0.293, 0.617, 1.0	0.0, 0.048, 0.191, 0.412, 0.691, 1.0

**Table 9:** RMS TRE results with edge virtual plane and equal spacing.

	8°	12°	16°	20°	Row Mean
2 nodes	1.095 mm	1.126 mm	0.971 mm	1.029 mm	1.055mm
3 nodes	0.981 mm	1.118 mm	0.929 mm	0.889 mm	0.979 mm
4 nodes	1.066 mm	0.961 mm	0.948 mm	1.028 mm	1.001 mm
5 nodes	1.053 mm	0.970 mm	0.829 mm	0.865 mm	0.929 mm
6 nodes	0.984 mm	0.852 mm	**0.804** mm	0.894 mm	**0.883** mm
Column Mean	1.036 mm	1.005 mm	**0.896** mm	0.941 mm	

The lowest values are highlighted in bold.

**Table 10:** RMS TRE results with edge virtual plane and half-cosine spacing.

	8°	12°	16°	20°	Row Mean
3 nodes	1.043 mm	1.092 mm	0.951 mm	0.957 mm	1.011 mm
4 nodes	1.020 mm	1.097 mm	0.905 mm	0.902 mm	0.981 mm
5 nodes	0.911 mm	0.890 mm	**0.762** mm	0.798 mm	**0.840** mm
6 nodes	0.953 mm	0.888 mm	0.919 mm	0.848 mm	0.902 mm
Column Mean	0.982 mm	0.992 mm	0.884 mm	**0.876** mm	

The lowest values are highlighted in bold.

During the hardware verification experiments, the Hall-effect sensors were sampled at a constant rate of 50 Hz, while optical tracking was performed at 100 Hz. Each sweep motion took an average of 3.16 s and yielded an average of 158 Hall-effect sensor samples. Sensor data processing and inference using the trained ANN models took 1.7 ms per pose estimate on a AMD Ryzen 7 5700X3D CPU. The total latency per pose estimate is dominated by the duration of the sensor apparatus’s sweep motion. Increasing the sweep speed would reduce the number of Hall-effect sensor samples collected, thereby increasing the tracking rate, potentially at the cost of accuracy.

### 3.2. 3D Slicer Virtual Environment

Hardware verification setup is also mirrored in the 3D Slicer virtual environment, as shown in Figure 9, for visualization and future surgical navigation purposes. The connection between the virtual and physical environments was established by streaming real-time transformation data to 3D Slicer via the OpenIGTLink protocol. Sub-millimeter registration results demonstrate a promising match between the virtual and physical environments, as shown in Figure 5.

**Figure 15:**
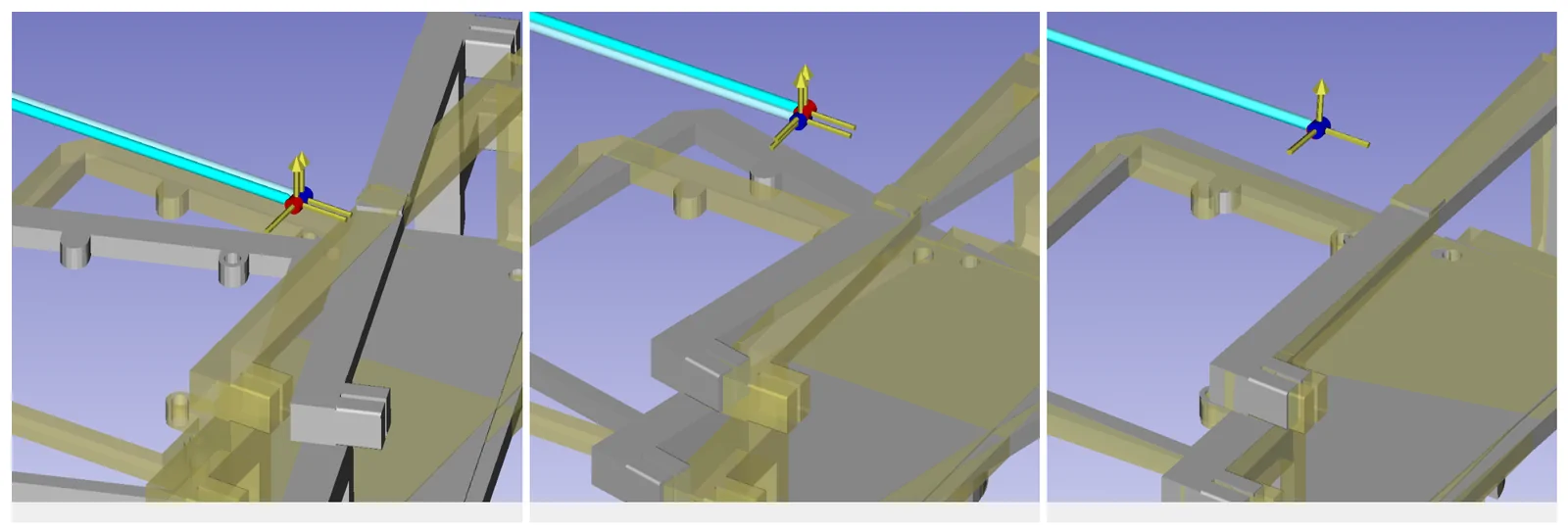
True microrobot pose (red) and estimated microrobot (blue) in 3D Slicer software.

The estimated microrobot pose is streamed into the constructed virtual environment as shown in Figure 15.

## 4. Discussion and Conclusions

In this study, a methodology is proposed to estimate the pose of a microrobot actuated within a prescribed enclosed workspace. The results reveal that the proposed methodology successfully estimates the pose of a 3 mm by 3 mm cylindrical microrobot by training two separate ANN models for position and orientation estimation. Registration procedures for the manufactured sensor apparatus and the robot manipulator were performed to enable efficient surgical navigation between the physical hardware and its virtual representations in a virtual environment. Pose data for the sensor apparatus collected from optical motion capture systems and data from the Hall-effect sensors are synchronized and fused via a developed ROS2 program to create virtual sensor readings with the desired arc degree, spacing method, and virtual plane placements. ANN models are trained using a comprehensive dataset of matching virtual sensor readings and microrobot poses, which were precisely controlled by the robot manipulator. All related hardware poses are streamed to a virtual environment created using 3D Slicer software. Microrobot pose estimation is achieved with a minimum RMS position TRE of 0.498 mm and a minimum RMS orientation error of 0.014 rad.

**Figure 16:**
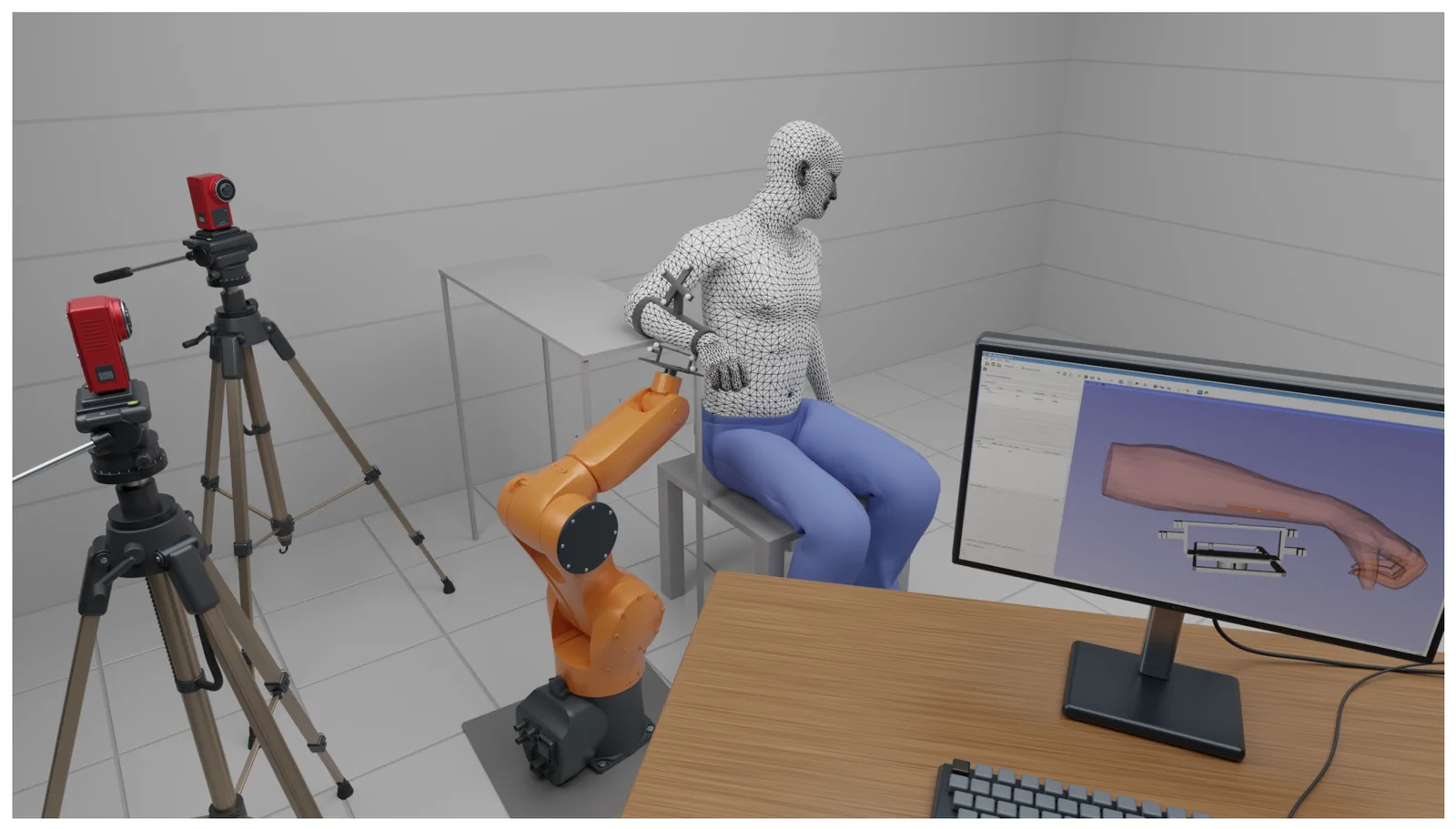
Illustration of the conceptual microrobot tracking system.

It should be noted that in real-world clinical scenarios, the microrobot will be actuated untethered within a larger closed anatomical workspace. Thus, to provide continuous tracking by increasing the sensing workspace of the Hall-effect sensor array, the sensor apparatus can be attached to the end effector of a robot manipulator. Consequently, without altering the proposed methodology, the microrobot’s pose can still be estimated using the established registration and calibration procedures. Additionally, to ensure efficient virtual plane placement, anatomical landmarks extracted from CT or MRI images along the planned microrobot path should be utilized to reduce the overall scanning workspace. As illustrated in Figure 16, the patient’s pre-scanned CT or MRI image can be registered via point-based registration or other suitable methods and can be optically tracked using a fixed virtual rigid body for patient motion compensation. The proposed use of a robot-mounted sensor apparatus and the optical tracking of the patient’s CT/MRI images are future directions that can expand the accurate tracking volume to the robot manipulator’s workspace while providing motion compensation for the patient.

The results presented here utilize mechanical actuation for the microrobot via the robot manipulator arm. In a practical system, the Hall-effect sensors would measure the actuation field in addition to the magnetic field of the microrobot. As a potential solution, actuation and localization can be decoupled by turning off the external field during localization. Since the sensor apparatus is optically tracked, the external field can be turned off as the sweep motion approaches the node positions specified in Table 5 and Table 8 and turned back on as the sensor apparatus travels to the next node position. While this does not reduce the tracking rate, any potential movement of the microrobot during the sweep between node positions could disrupt the ANN pose estimation. Alternatively, the external field could remain off during the entire sweep motion, sacrificing the tracking rate as the sweep movement is paused during actuation.

In the hardware verification experiments, the sensing depth is limited to 30 mm. With the current proof-of-concept microrobot size (a 3 mm by 3 mm cylindrical magnet), pose estimation performance begins to degrade beyond a 30 mm sensing depth, as shown in Figure 14. Microrobot size could be reduced by using a higher-grade NdFeB magnet and/or higher-sensitivity Hall-effect sensors.

## Figures and Tables

**Table 1:** Comparison of Hall-effect sensor-based microrobot localization methods.

	Microrobot Size, Shape and Type	Sensing Depth	Estimated DoFs	Tracking Period (Rate)	Number of Hall-Effect Sensors (Model)	Pose Prediction Error Achieved
[8]	6.4 mm by 6.4 mm by 12.8 mm cuboid magnet	5 mm–50 mm	5 (3 translation, 2 rotation)	0.005 s (200 Hz)	64 (A1389)	2.1 mm mean
[6]	1 mm by 1 mm cylinder NdFeB magnet with 4 mm long helical copper spring	5 mm	1 (translation along the vein)	not reported	16 (TLV493D-A1B6)	2.35 mm mean
[5]	5 mm cubic NdFeB magnet	4 mm and 10 mm	2 (2 translation)	3.5 s (0.28 Hz)	9 (ALS31300)	0.047 mm mean
[20]	0.75 mm by 1 mm cylindrical N50-grade NdFeB magnet	20 mm	3 (3 translation)	1 s (1 Hz)	4 (MLX90393)	1.5 mm mean
The proposed method	3 mm by 3 mm cylindrical N35-grade NdFeB magnet	30 mm	4 (2 translation, 2 rotation)	3.16 s (0.32 Hz)	4 (EQ730L)	0.498 mm RMS

**Table 2:** Utilized hardware and their respective calibration/registration methods.

Hardware	Calibration/Registration Methodology	Utilized Software (Version)
Optitrack Flex 3 Cameras	Wanding Process	Motive Tracker (1.7.2)
Stylus Tool	Spin and Pivot Calibration	Slicer 3D (5.10.0) [26] SlicerIGT (d0eb332) [27]
Robot Manipulator	Robot Registration [23]	Custom Software
Sensor Apparatus	Rigid Body Registration	Slicer 3D (5.10.0) SlicerIGT (d0eb332)
Sensor Apparatus	ANN	Scikit-Learn (1.9.1)

**Table 3:** Scan arc length effect on usable 3D scanning volume of the sensor apparatus.

	8°	12°	16°	20°
Virtual plane at middle	37°	33°	29°	25°
Virtual plane at edge	45°	45°	45°	45°

**Table 4:** Utilized hardware and respective calibration/registration error.

Hardware	Calibration/Registration Methodology	Utilized Software	Calibration/Registration Error
Optitrack Flex 3 Cameras	Wanding Process	Motive Tracker	0.058 mm mean
Stylus Tool	Spin and Pivot Calibration	Slicer 3D SlicerIGT	0.199 mm RMS
KUKA KR6 R900 SIXX	Robot Registration	Custom Software	0.121 mm RMS (FRE)
Sensor Apparatus	Rigid Body Registration	Slicer 3D SlicerIGT	0.294 mm RMS (FRE)
Sensor Apparatus	ANN	Scikit-Learn	Table 5, Table 6, Table 7, Table 8, Table 9 and Table 10

## Data Availability

The original contributions presented in this study are included in the article/Supplementary Materials. Further inquiries can be directed to the corresponding author.

## Supplementary Materials

The following supporting information can be downloaded at: https://www.mdpi.com/article/10.3390/mi17101196/s1.
